# Bending and Scission: When the Membraneless Condensates Meet Endosome Membrane

**DOI:** 10.1002/mco2.70165

**Published:** 2025-04-01

**Authors:** Zhengkun Zhang, Feng Xie, Long Zhang

**Affiliations:** ^1^ Department of Radiation Oncology and the State Key Laboratory of Transvascular Implantation Devices the Second Affiliated Hospital Zhejiang University School of Medicine Hangzhou China; ^2^ The First Affiliated Hospital the Institutes of Biology and Medical Sciences Suzhou Medical College Soochow University Suzhou China; ^3^ The MOE Basic Research and Innovation Center for the Targeted Therapeutics of Solid Tumors The First Affiliated Hospital Jiangxi Medical College Nanchang University Nanchang China

1

In a recent study reported in *Nature*, Xiaofeng Fang's group from Tsinghua University and a team led by Roland Knorr from the University of Cologne in Germany discovered that the plant protein FREE1 can form condensates through phase separation, which drive endosomal membrane invagination and instability via wetting phenomenon [1]. This study highlights biomolecular condensates' critical role in physiological processes, particularly in mediating endosomal sorting complex required for transport (ESCRT) and adenosine triphosphate (ATP) independent intraluminal vesicle (ILV) formation.

Multivesicular bodies (MVBs) are vital organelles within cells that are primarily responsible for delivering cargo molecules from the endocytic pathway to lysosomes for degradation and recycling and for regulating biological processes such as nutrient uptake, immunity, and signal transduction [2]. The MVB membrane forms ILVs through invagination and scission; these ILVs then sort protein cargo and require the consumption of ATP by the ESCRT protein complex. Biomolecular condensates result from liquid–liquid phase separation (LLPS) and typically exhibit the characteristics of liquid droplets or gel‐like aggregates, performing various functions within cells. Previous studies have shown that condensates interact with membranes, leading to “wetting” and capillary phenomena. However, the biological significance of wetting‐related capillary forces in cellular processes remains largely unknown [3]. In the study led by Xiaofeng Fang and colleagues, the authors used in vitro reconstitution, computer simulations, and genetic experimental analysis to discover that FREE1 condensates can induce membrane curvature and invagination independently of the ESCRT protein complex and ATP. Notably, the ATP‐independent membrane scission mediated by FREE1 condensates was primarily supported by in vitro reconstitution and computer simulations, whereas the genetic experimental analysis (e.g., vps2.1 knockout complementation) indirectly supports ESCRT‐independence. The study first used biotinylated isoxazole (b‐isox) compounds to precipitate and screen proteins with phase‐separation capabilities [4], revealing that FREE1 possesses robust phase‐separation abilities both in vivo and in vitro. This phase separation ability is unaffected by salt concentration, and the intrinsically disordered region (IDR) at the N‐terminus of FREE1 is required for phase separation. In addition, FREE1 contains an FYVE domain capable of binding to the membrane lipid phosphatidylinositol 3‐phosphate (PI3P), enabling its localization to the MVB membrane. Further research revealed that the formation of condensates significantly enhances the membrane‐binding capacity of FREE1. Moreover, FREE1 condensates served as scaffolds, recruiting other ESCRT components, particularly the ESCRT‐I subcomplex (including VPS23, VPS28, and VPS37), as client molecules into their condensates.

In vitro reconstitution experiments revealed that small membrane vesicles filled with FREE1 condensates freely diffused inside giant unilamellar vesicles (GUVs). When GUVs containing PI3P were exposed to the purified FREE1 protein, numerous micrometer‐sized FREE1 condensates were formed on the GUV surface. These FREE1 condensates interacted with the membrane through capillary forces, causing membrane deformation and invagination and resulting in structures resembling ESCRT‐mediated ILVs. This suggests that the FREE1 condensates alone are sufficient to mediate vesicle scission. Theoretical calculations support this hypothesis. To further confirm that phase separation is required for FREE1 to function normally, the IDR of FREE1 was replaced with FUS‐IDR, which has a completely different sequence but retains its phase separation ability, or FUS‐IDRm, which lacks phase separation ability. They found that only FUS‐IDR, which possesses phase separation ability, fully complemented the lethal phenotype of FREE1 mutants, whereas FUS‐IDRm did not. In addition, although FUS‐IDR‐FREE1 could substitute for FREE1, it could not interact with ESCRTs to recruit them into their condensates; this implies that FREE1 condensates can function independently of ESCRTs. However, while FUS‐IDR‐FREE1 supported plant growth and development under normal conditions, it did not meet the germination and survival rates of plants under osmotic stress caused by high salt and drought conditions. This suggests that the ESCRT machinery and FREE1 condensates form a dual insurance mechanism for MVB biogenesis that has been preserved during evolution.

This study unambiguously elucidated the biological significance of wetting‐related capillary forces between condensates and membranes in cellular processes, uncovering a novel mechanism for promoting the production of MVBs that are independent of ESCRT machinery and ATP mediation. Condensation with liquid‐like properties formed through FREE1 phase separation drives the invagination of MVB membranes through wetting, leading to instability at the membrane neck and ultimately completing membrane scission to form ILVs (Figure 1). However, this study primarily focused on in vitro reconstitution and cellular‐level analyses; while experiments included Arabidopsis seedlings, the physiological relevance of FREE1‐mediated membrane scission in intact plants requires further validation. Direct experimental evidence linking FREE1 condensates to internalization of ubiquitinated cargo via ILV formation remains to be established. In addition, other potential regulatory mechanisms and downstream effects have not been thoroughly explored, and more biophysical methods should be used to conduct in‐depth analyses of the kinetics and structural characteristics of FREE1 phase separation.

**FIGURE 1:**
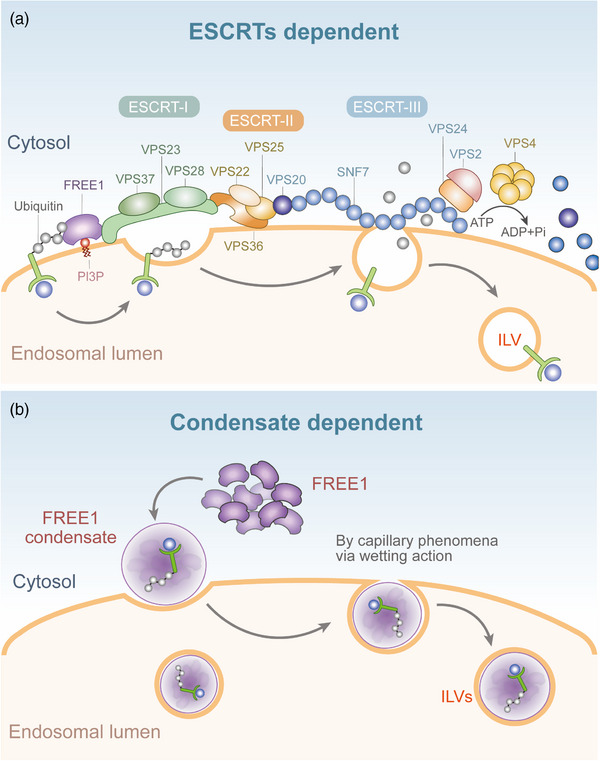
A model depicting the formation pathways of endosomal sorting complex required for transport (ESCRT)‐machinery‐dependent and FREE1‐condensate‐mediated intraluminal vesicles (ILVs) originating from multivesicular body (MVB) membranes (*orange*). (a) Typically, in the ESCRT‐machinery mechanism, ESCRT‐0 initiates its function first. However, in plants, ESCRT‐0 and its homologous protein components are absent. Nevertheless, there are other protein components that possess similar functionalities. Among these, FREE1 can bind to phosphatidylinositol 3‐phosphate (PI3P) to move to the multivesicular MVB^2^ while simultaneously recruiting the ESCRT‐I subunit, which subsequently recruits ESCRT‐II to assemble into a supramolecular complex. This complex induces the deformation of the endosome membrane, gathering the sorted cargo proteins at the budding site. ESCRT‐II then recruits ESCRT‐III, promoting the polymerization of ESCRT‐III components into filamentous spirals that further tighten the “neck” region of the invaginating vesicle. Finally, the Vps4 complex induces the disassembly of the ESCRT‐III complex, facilitating the scission of the invaginated vesicle, which then detaches from the membrane. (b) Within the condensate mechanism, FREE1 proteins form condensates with ubiquitinated cargoes, spontaneously inducing membrane invagination and scission, ultimately resulting in the formation of ILVs. ESCRT, endosomal sorting complex required for transport; ILVs, intraluminal vesicles; PI3P, phosphatidylinositol 3‐phosphate.

This study revealed a new mechanism mediated by biomolecular condensates, which not only enhances our understanding of the dynamic processes of intracellular membranes but also provides new insight into the molecular mechanisms underlying plant tolerance to environmental osmotic stress. Membrane vesicle formation and membrane remodeling play pivotal roles in the onset and progression of numerous diseases, such as neurodegenerative disorders and cancers. The newly discovered mechanism in this research may offer novel targets or approaches for the diagnosis and treatment of related diseases. It also suggests that biological condensates play crucial roles in numerous physiological functions that have previously been overlooked. This urges subsequent scientific research to focus on the biological significance of condensates and their interactions with other cellular components. Future studies should introduce more experimental methods and techniques to further investigate the functions of condensates.

## Author Contributions

Zhengkun Zhang wrote the manuscript and prepared the figure. Feng Xie provided valuable discussion. Long Zhang approved the final version of the manuscript. All authors have read and approved the final manuscript.

## Ethics Statement

The authors have nothing to report.

## Conflicts of Interest

The authors declare no conflict of interest.

## Data Availability

The authors have nothing to report.
